# Effects of Inflammation and Depression on Telomere Length in Young Adults in the United States

**DOI:** 10.3390/jcm8050711

**Published:** 2019-05-19

**Authors:** Dayeon Shin, Jungwoon Shin, Kyung Won Lee

**Affiliations:** 1 Department of Food and Nutrition, Inha University, Incheon 22212, Korea; dyshin@inha.ac.kr; 2 Department of Computer Science and Engineering, Korea University, Seoul 02841, Korea; jungwoonshin@gmail.com; 3 Division of Epidemiology and Health Index, Center for Genome Science, Korea National Institute of Health, Korea Centers for Disease Control and Prevention, Chungcheongbuk-do 28160, Korea

**Keywords:** inflammation, C-reactive protein, depression, telomere length, National Health and Nutrition Examination Survey (NHANES)

## Abstract

Little is known about the associations of inflammation and depression with telomere length. Using data from the National Health and Nutrition Examination Survey (NHANES) 1999–2002, the current study assessed the effects of inflammation and depression on telomere length in 1141 young adults in the USA. Depression status was assessed from the World Health Organization Composite International Diagnostic Interview and inflammation status was measured based on C-reactive protein (CRP) concentrations. Information on telomere length was obtained using the quantitative polymerase chain reaction method to measure telomere length relative to standard reference DNA (T/S ratio). Unadjusted and adjusted linear and logistic regression models were used to assess the relationship between the tertiles of CRP concentration and the telomere length stratified by the status of depression such as major depression or depressed affect vs. no depression. The adjusted models were controlled for age, family poverty income ratio, race/ethnicity, marital status, physical activity, body mass index, and alcohol drinking status. A significant and decreasing linear trend in telomere length was found as CRP levels increased in men, regardless of the depression status, and women with major depression or depressed affect (*p* values < 0.05). Among men without depression, those with an elevated CRP level had increased odds of having a shortened telomere length compared to men with low CRP levels after controlling for covariates (adjusted odds ratio 1.77, 95% confidence interval (CI) 1.09–2.90). In women, there was no association between CRP and telomere length, regardless of the depression status. In conclusion, there was a significant and inverse association between inflammation and telomere length according to the depression status in men but not in women. The present findings may be of clinical significance for the monitoring of inflammation levels and depression status as determinants of telomere length.

## 1. Introduction

Telomeres are DNA‒protein complexes composed of base pairs of TTAGGG repeats at the end of linear chromosomal DNA [1], which protect the DNA from damage [2]. Telomeres shorten with cell replication and cell division [3] and reflect cell turnover. In adults, shortened leukocyte telomere length reflecting the different lengths of telomeres at the ends of the 23 chromosomes [4], has been suggested as a robust biomarker related to biological aging [5,6,7,8,9]. Short telomere length has been associated with increased risks for morbidity and mortality [10].

One of the major proposed factors for shortened telomere length is mental health, specifically the status of depression [11,12,13,14,15], although mixed findings on the relationship between depression and telomere length have been reported in cross-sectional and prospective cohort studies [16,17,18,19]. In a general Danish population study, short telomere length was not associated with an increased risk of depression [19]. A large-scale population-based study demonstrated no association between telomere length and depression, whereas a relatively small sample-based study reported an inverse relationship between telomere length and depression. These inconsistent findings may be due to the presence of chronic inflammation, which influences both depression and telomere length.

Depression is commonly associated with suicidal behavior and medical illnesses related to a further enhanced suicide risk [20]. In particular, medical disorders have been identified as a significant risk factor for both suicidal behavior and suicidal ideation, especially among depressed patients [20]. Short telomere length may be involved not only in depression but also in determining the increased rate of both medical disorders and suicidality. Specifically, the existence of a previous mood disorder, prior and current history of medical disorders, and cognitive impairment were reported to be the most important risk factors for suicide. 

Previous research on the mechanisms of telomere erosion identified inflammation as having an important influence on telomere length [21,22]. In particular, chronic inflammation has been suggested to influence overall cell turnover and is associated with a shorter telomere length [23] as well as an increased risk for depression [24]. However, limited knowledge exists on the combined effects of inflammation and depression on telomere length in young adults. 

Therefore, in the present study, we hypothesized that there are different combined effects of inflammation and depression status on telomere length in young adults in the USA. The aim of this study was to investigate the interrelationships among inflammation, depression, and telomere length using a nationally representative sample of adults in the USA.

## 2. Materials and Methods

### 2.1. Dataset

Data were obtained from the National Health and Nutrition Examination Survey (NHANES) 1999–2002 because telomere length data were only available for these specific years. All participants aged 20 years and over had blood samples collected for DNA purification. Data on telomere health and diagnosis of depression were available for 1292 adults. Of those, 151 participants with missing information on education, marital status, family income ratio, and body mass index (BMI) were excluded. The final analytic sample for this study was 1141 adults (496 men and 645 women). NHANES is a publicly available dataset that can be downloaded from the NHANES website (http://www.cdc.gov/nchs/nhanes.htm). The NHANES protocols were approved for NHANES 1999–2002 by the National Center for Health Statistics Research ethics review board, written informed consent was obtained for all participants [25], and additional Institutional Review Board approval for the secondary analyses was not required for this study.

### 2.2. C-Reactive Protein

C-reactive protein (CRP) is an acute phase response to an infectious disease or other cause of tissue damage and inflammation. CRP level was measured by latex-enhanced nephelometry [26].

### 2.3. Depression

In NHANES, three modules (generalized anxiety disorder, major depression, and panic disorder) from the automated version of the World Health Organization Composite International Diagnostic Interview, Version 2.1 (CIDI-Auto 2.1), assessed over the past 12 months, were administered during the face-to-face portion of the Mobile Examination Center (MEC) interview. The CIDI is a fully standardized interview according to the definitions and criteria of the 10th revision of the International Classification of Diseases (ICD) [27] and the fourth edition of the American Psychiatric Association’s Diagnostic and Statistical Manual of Mental Disorders (DSM-IV) [28]. The instrument was administered to a half-sample of participants aged from 20 to 39 years who had received a physical examination.

For the present study, information obtained from the CIDI was then categorized as (1) major depression or depressed affect; or (2) no depression. Major depression was assessed based on the diagnostic criteria of the ICD-10 and DSM-IV. Participants who had periods of feeling sad, depressed, or empty for two weeks or longer but who did not meet the diagnostic criteria for major depression were categorized as having a depressed affect.

### 2.4. Telomere Length

All participants aged 20 years and older examined in 1999–2000 or in 2001–2002 who had blood collected for DNA purification were eligible. Blood samples were obtained from participants of the NHANES surveys. A small portion of the blood samples was stored for later analysis of DNA samples. The telomere length assay was performed in the laboratory of Dr. Elizabeth Blackburn at the University of California, San Francisco, using the quantitative polymerase chain reaction (PCR) method to measure telomere length relative to standard reference DNA (T/S ratio), as described in detail elsewhere [29,30].

The conversion from T/S ratio to base pairs was calculated based on a comparison of telomeric restriction fragment (TRF) length from Southern blot analysis and T/S ratios using DNA samples from the human diploid fibroblast cell line IMR90 at different population doublings [31]. 

### 2.5. Statistical Analyses

Descriptive statistics were used to assess the overall distribution of sociodemographic and lifestyle factors by the tertiles of telomere length. Chi-square tests were used to detect differences in categorical variables by the tertiles of CRP. One-way analysis of variance (ANOVA) was performed to examine the differences in continuous variables by the tertiles of CRP. The geometric mean of telomere length was calculated in relation to the tertiles of CRP. Testing for linear trends was conducted by assigning the median values for telomere length to each tertile of CRP and indicating each tertile in the model with recorded ordinal variables. Unadjusted and adjusted logistic regression models were used to assess the relationship between CRP concentrations (≤0.2 vs. >0.2 mg/dL) and shortened telomere length stratified by the depression status (no depression vs. major depression or depressed affect). Shortened telomere length was defined as a telomere length less than the median value. Adjusted models were controlled for age (continuous), family poverty income ratio (PIR) (continuous), race/ethnicity (Mexican-American or other Hispanic, non-Hispanic white, non-Hispanic black, other race including multi-racial), marital status (never married, married or living with partner, widowed/divorced/separated), physical activity (0 metabolic equivalent (MET), 0–500 MET, 500–1000 MET, >1000 MET), and BMI (<25 vs. ≥25 kg/m^2^). Because telomere length and CRP were skewed, the values were log-transformed. Linear regression models were performed between log-transformed telomere length and log-transformed CRP according to the depression status and sex.

In men, age, BMI, CRP, and marital status differed significantly according to the tertile of telomere length (*p* values < 0.05). In women, age, family PIR, and alcohol status differed significantly by the tertile of telomere length (*p* values < 0.05) (Table 1). Age, BMI, and CRP were highest in men in the lowest tertile for telomere length. Age and family PIR were highest in women in the lowest tertile for telomere length.

A significant and decreasing trend in telomere length was observed in men both with and without depression as the CRP concentration moved from tertile 1 to tertile 3 (*p* values < 0.05). In women with or without depression, a significantly decreasing trend in telomere length was also observed as the CRP concentration moved from tertile 1 to tertile 3 (*p* values < 0.05) (Table 2).

In the unadjusted models, men with CRP concentrations >0.2 mg/dL had increased odds of having short telomere length compared to the odds in men with CRP concentrations ≤0.2 mg/dL (odds ratio (OR) 2.24, 95% CI 1.39–3.59). In the adjusted model, the significant association between elevated CRP concentration and short telomere length remained in men without depression (adjusted OR (AOR) 1.81, 95% CI 1.12–2.91). However, there was no significant association between depression status and short telomere length in either men or women (Table 3).

The combined effects of CRP concentration and depression status on shortened telomere length by sex were examined. Men without depression with elevated CRP concentration (>0.2 mg/dL) had increased odds of having a shortened telomere length in both unadjusted and adjusted models (OR 2.16, 95% CI 1.37–3.39; AOR 1.77, 95% CI 1.09–2.90). In contrast, there was no significant association between CRP concentration and telomere length in men with depression. No association was found between CRP concentration and telomere length in women, regardless of the presence of depression (Table 4).

**Figure 1:**
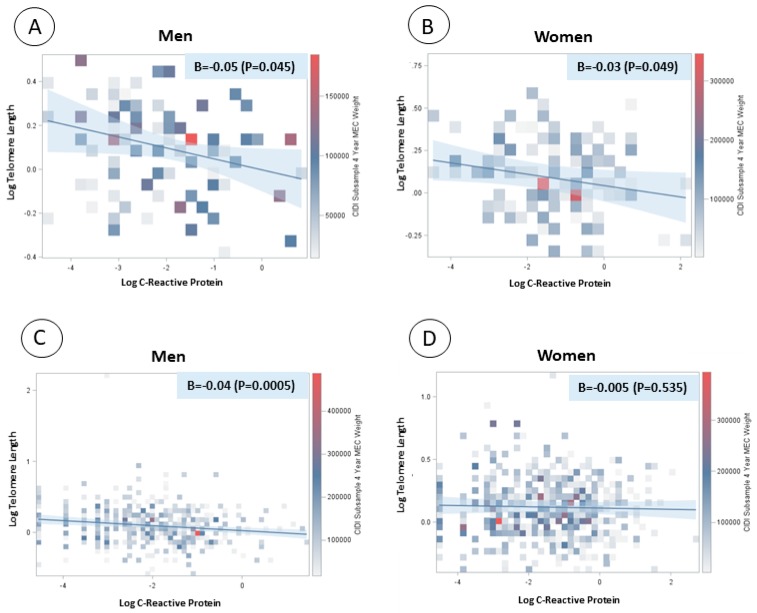
Linear regression models between log-transformed C-reactive protein and log-transformed telomere length by sex and depression status. (**A**) Men with major depression or depressed affect; (**B**) women with major depression or depressed affect; (**C**) men without depression; and (**D**) women without depression.

There was a significant and inverse linear association between log-transformed CRP concentration and log-transformed telomere length in both men and women with major depression or depressed affect (β = −0.05, *p* value = 0.045; β = −0.03, *p* value = 0.049, respectively). There was also a significant and inverse association between log-transformed CRP concentration and log-transformed telomere length in men without depression (β = −0.04, *p* value = 0.0005) but not in women without depression (Figure 1).

## 3. Discussion

In the present study, we observed a significant association between elevated levels of inflammation and shortened telomere length only in men without depression. We did not observe this relationship in men with major depression or depressed affect or in women regardless of the depression status. Consistent with our findings, Needham et al. [17] reported a lack of association between depression, anxious symptomology, and telomere length in young adults across different racial and ethnic groups using the NHANES 1999–2002. The authors indicated that the lack of association might have been due to the fact that telomere length reflects biological aging in later life, rather than in the early stages of life. This explanation can be applied to our findings of a lack of associations between CRP and telomere length in both men and women with depression.

People become less resilient to stress because physiological aging may modify the responsivity to stress [32,33]. Although the underlying mechanisms still need to be investigated, this might be partially explained by the kynurenine pathway, which is part of tryptophan catabolism [34]. As the kynurenine pathway is activated, tryptophan levels decrease, which may lead to depression [35] and age-related neurodegenerative diseases [36]. This was also supported by a previous animal study using female Wistar rats that indicated significant changes in the kynurenine pathway with increasing age [37]. Furthermore, higher accumulation levels of damaged tissues and cells were observed in advanced age. Such oxidized proteins are more likely to be attacked by free radicals, and this may contribute to oxidative stress [38]. Therefore, participants aged 20–39 years may be resilient to stress, which was not indicated by shortened telomere length in our study.

In another study by Shaffer et al. [18], the authors suggested that the absence of an association between concurrent depressive symptoms and telomere length may reflect the fact that leukocyte telomere length is influenced by cumulative environmental factors over time, whereas assessments of depressive symptoms generally focus on only the past 1–2 weeks. This explanation could also apply to our study findings, since telomere length reflects a long period of time, whereas the survey assessed depression status over the past 12 months.

In this study, a linear regression model revealed that telomere length decreased as CRP levels increased in men, regardless of the depression status, and in women with major depression or depressed affect, but not in women without depression. This illustrated an inverse linear relationship between telomere length and inflammation in young adults in the USA, except for women without depression. Similar results were observed for multivariable logistic analyses. Among men without depression, those with higher levels of inflammation (CRP > 0.2 mg/dL) had increased odds of having shortened telomere length compared to those in men with lower levels of inflammation (CRP ≤ 0.2 mg/dL). In contrast, there was no association between CRP, depression status, and telomere length in women.

Contrasting findings to those of the present study could be partially explained by sex differences due to hormonal responses, estrogen vs. testosterone, and antioxidant capacity [33,34] between men and women. Testosterone increases susceptibility to oxidative stress [39], whereas estrogen has been suggested to activate telomerase [40] and might provide protection to telomeres [41], antagonizing the effect of telomere shortening during early stages of life [42]. In addition, previous studies reported significant associations between fluctuations in estrogen levels and the risk of depression [43,44] and decreased estrogen level in women experiencing depression [45]. While estrogen may have telomere protection activity among women without depression, this may not have been the case in the women with major depression or depressed affect in the current study. The present study has several strengths and limitations. Firstly, this study used a racially diverse and nationally representative sample of young adults in the USA; thus, the findings may be generalizable to the young adult population in the USA. Depression was assessed using the CIDI, a reliable and structured instrument [46]. The study controlled for important confounders including age, family PIR, race/ethnicity, marital status, physical activity, and BMI. Despite the strengths of this study, there are also limitations. Telomere length was measured in leukocytes, which did not reflect telomere length in other cells or tissues. Furthermore, no measurement of telomerase activity was available in this study. High telomerase activity with short telomere length has been associated with distress in early life and impaired psychosocial resources [42]. Lastly, this research was limited by its cross-sectional design and subsequent inability to address questions of causality regarding how the depression status influences the association between inflammation and telomere length. Future research is warranted to provide insights into the associations between depression, inflammation, and telomere shortening through prospective cohort studies.

In conclusion, there was a significant and inverse linear association between inflammation and telomere length in men with and without depression and women with depression. The associations of inflammation and depression with telomere length differed by sex in young adults in the USA. Among men without depression, those with an elevated CRP level had increased odds of having a shortened telomere length compared to men with low CRP levels after controlling for covariates. Finally, in women, there was no association between CRP and telomere length, regardless of the depression status. The present findings may be of clinical significance for the monitoring of inflammation levels and depression status as determinants of telomere length.

## Figures and Tables

**Table 1:** Sociodemographic and lifestyle characteristics across the tertiles of telomere length.

	Men							Women						
	Telomere Length							Telomere Length						
	Tertile 1 (*n* = 165)		Tertile 2 (*n* = 166)		Tertile 3 (*n* = 165)			Tertile 1 (*n* = 215)		Tertile 2 (*n* = 215)		Tertile 3 (*n* = 215)		
	Mean	(SEM)	Mean	(SEM)	Mean	(SEM)	*p* Value	Mean	(SEM)	Mean	(SEM)	Mean	(SEM)	*p* Value
**Age**	31.8	(0.5)	29.2	(0.6)	28.5	(0.6)	<0.0001	31.3	(0.6)	29.5	(0.5)	28.9	(0.6)	0.0062
**Family poverty income ratio**	2.9	(0.2)	2.9	(0.2)	2.7	(0.1)	0.0925	2.9	(0.1)	2.8	(0.1)	2.2	(0.1)	0.0006
**BMI (kg/m^2^)**	28.4	(0.5)	27.7	(0.5)	25.6	(0.4)	<0.0001	27.5	(0.7)	26.6	(0.6)	27.2	(0.5)	0.7436
**C-reactive protein (mg/dL)**	0.33	(0.04)	0.29	(0.04)	0.20	(0.03)	0.027	0.46	(0.07)	0.43	(0.03)	0.41	(0.05)	0.5535
	***n***	**(Wt’d %)**	***n***	**(Wt’d %)**	***n***	**(Wt’d %)**	***p* value**	***n***	**(Wt’d %)**	***n***	**(Wt’d %)**	***n***	**(Wt’d %)**	***p* value**
**Race/Ethnicity**														
Mexican-American or other Hispanic	66	(19.4)	56	(16.1)	47	(13.2)	0.5876	69	(18.5)	78	(18.1)	56	(16.5)	0.8552
Non-Hispanic white	72	(68.2)	75	(68.0)	83	(73.0)		103	(68.0)	98	(67.8)	101	(65.3)	
Non-Hispanic black	21	(7.0)	29	(9.9)	30	(9.7)		34	(9.9)	32	(10.6)	48	(14.9)	
Other-including multi-racial	6	(5.5)	6	(6.0)	5	(4.1)		9	(3.6)	7	(3.5)	10	(3.3)	
**Education**														
≤11th grade	53	(21.6)	51	(21.1)	43	(17.8)	0.3973	54	(15.7)	50	(17.0)	49	(16.8)	0.9493
High school grad/GED or equivalent	43	(28.5)	38	(25.6)	54	(35.2)		49	(27.4)	53	(24.3)	50	(23.3)	
≥ Some college or associate’s degree	69	(49.9)	77	(53.3)	68	(47.0)		112	(56.9)	112	(58.8)	116	(59.9)	
**Marital status**														
Never married	38	(23.2)	75	(44.4)	74	(43.8)	0.0006	37	(20.4)	55	(28.5)	71	(36.3)	0.0623
Married/living with partner	114	(69.4)	78	(47.3)	82	(50.7)		147	(65.5)	141	(61.3)	121	(51.5)	
Widowed/divorced/separated	13	(7.4)	13	(8.2)	9	(5.5)		31	(14.1)	19	(10.2)	23	(12.2)	
**Depression**														
Major depression or depressed affect	24	(13.7)	30	(19.0)	29	(15.2)	0.5171	40	(19.9)	35	(17.8)	37	(17.9)	0.924
No	141	(86.3)	136	(81.0)	136	(84.8)		175	(80.1)	180	(82.2)	178	(82.1)	
**Physical activity**														
0 MET	64	(32.0)	55	(29.1)	49	(29.4)	0.5099	97	(37.0)	78	(28.5)	78	(25.3)	0.1379
0–500 MET	34	(24.3)	40	(22.1)	32	(17.2)		46	(22.4)	53	(23.5)	57	(30.3)	
500–1000 MET	12	(7.6)	13	(7.3)	21	(12.4)		30	(17.1)	26	(12.9)	26	(14.2)	
>1000 MET	55	(36.2)	58	(41.5)	63	(41.0)		42	(23.5)	58	(35.1)	54	(30.2)	
**Smoking status**														
Never	87	(51.7)	95	(58.1)	73	(43.7)	0.0637	135	(52.7)	143	(61.9)	135	(59.4)	0.4439
Past	25	(14.9)	11	(7.1)	29	(16.9)		41	(18.2)	24	(11.8)	25	(9.8)	
Current	53	(33.5)	60	(34.8)	63	(39.4)		39	(29.1)	48	(26.3)	55	(30.9)	
**Alcohol status (*n* = 464 for men and *n* = 576 for women)**														
Lifetime abstainer	10	(5.9)	15	(8.0)	13	(10.4)	NA	31	(11.5)	48	(17.5)	53	(26.8)	0.0142
Former drinker	5	(4.1)	0	(0.0)	1	(1.3)		10	(3.5)	5	(1.5)	4	(1.7)	
Current drinker with moderate alcohol consumption	59	(40.5)	55	(38.0)	48	(30.4)		60	(29.4)	45	(22.5)	44	(21.9)	
Current drinker with above moderate alcohol consumption	75	(49.5)	89	(54.0)	94	(57.9)		95	(55.6)	92	(58.5)	89	(49.6)	

SEM: Standard error of the mean; NA: Not available; Wt’d %: Weighted %; GEM: General Education Development; MET: Metabolic equivalent.

**Table 2:** Geometric means of telomere lengths stratified by depression status and tertiles of C-reactive protein level by sex.

		C-Reactive Protein (mg/dL)						
		Tertile 1		Tertile 2		Tertile 3		*p*-for-Trend
		Geometric mean telomere length	(SEM)	Geometric mean telomere length	(SEM)	Geometric mean telomere length	(SEM)	
Men	No depression (*n* = 413)	1.15	(0.03)	1.12	(0.02)	1.04	(0.02)	<0.0001
	Major depression or depressed affect (*n* = 83)	1.17	(0.06)	1.14	(0.04)	1.01	(0.04)	<0.0001
Women	No depression (*n* = 533)	1.14	(0.04)	1.12	(0.02)	1.12	(0.03)	0.0074
	Major depression or depressed affect (*n* = 112)	1.15	(0.05)	1.06	(0.06)	1.07	(0.05)	<0.0001

SEM: Standard error of the mean. *p*-for-trend was obtained by applying the median values of each tertile group.

**Table 3:** Independent effects of C-reactive protein and depression with short telomere length.

	**Men**		**Women**	
	**C-Reactive Protein**		**C-Reactive Protein**	
Short telomere length	≤0.2 mg/dL	>0.2 mg/dL	≤0.2 mg/dL	>0.2 mg/dL
Unadjusted model	1.00 (Ref.)	2.24 (1.39–3.59)	1.00 (Ref.)	1.16 (0.83–1.62)
Multivariable model *	1.00 (Ref.)	1.81 (1.12–2.91)	1.00 (Ref.)	1.02 (0.69–1.52)
	Depression status		Depression status	
Short telomere length	No depression	Major depression or depressed affect	No depression	Major depression or depressed affect
Unadjusted model	1.00 (Ref.)	1.09 (0.71–1.67)	1.00 (Ref.)	0.94 (0.48–1.82)
Multivariable model *	1.00 (Ref.)	1.10 (0.72–1.66)	1.00 (Ref.)	0.74 (0.41–1.34)

* Adjusted for age (continuous), family poverty income ratio (continuous), race/ethnicity (Mexican-American or other Hispanic, non-Hispanic white, non-Hispanic black, other race including multi-racial), marital status (never married, married or living with partner, widowed/divorced/separated), physical activity (0 metabolic equivalent of task (MET), 0–500 MET, 500–1000 MET, >1000 MET), BMI (<25 kg/m^2^ vs. ≥25 kg/m^2^), and alcohol drinking (none, former drinker, current drinker). Shortened telomere length was defined as a telomere length less than the median value of telomere length. Ref.: Reference.

**Table 4:** The combined effects of inflammation and depression status on shortened telomere length by sex.

**Short Telomere Length**	**Depression Status**			
	**Major Depression or Depressed Affect**		**No Depression**	
	**C-Reactive Protein**		**C-Reactive Protein**	
Men	≤0.2 mg/dL	>0.2 mg/dL	≤0.2 mg/dL	>0.2 mg/dL
Unadjusted model	1.00 (Ref.)	2.68 (0.81–8.84)	1.00 (Ref.)	2.16 (1.37–3.39)
Multivariable model *	1.00 (Ref.)	0.94 (0.22–4.01)	1.00 (Ref.)	1.77 (1.09–2.90)
	**Major depression or depressed affect**		**No depression**	
	**C-reactive protein**		**C-reactive protein**	
Women	≤0.2 mg/dL	>0.2 mg/dL	≤0.2 mg/dL	>0.2 mg/dL
Unadjusted model	1.00 (Ref.)	2.23 (0.97–5.11)	1.00 (Ref.)	1.01 (0.65–1.56)
Multivariable model *	1.00 (Ref.)	2.56 (0.76–8.61)	1.00 (Ref.)	0.87 (0.52–1.43)

* Adjusted for age (continuous), family poverty income ratio (continuous), race/ethnicity (Mexican-American or other Hispanic, non-Hispanic white, non-Hispanic black, other race including multi-racial), marital status (never married, married or living with partner, widowed/divorced/separated), physical activity (0 metabolic equivalent of task (MET), 0–500 MET, 500–1000 MET, >1000 MET), BMI (<25 kg/m^2^ vs. ≥25 kg/m^2^), and alcohol drinking (none, former drinker, current drinker). Shortened telomere length was defined as a telomere length less than the median value of telomere length. Ref.: Reference.
